# Chemogenetic Modulation of Preoptic *Gabre* Neurons Decreases Body Temperature and Heart Rate

**DOI:** 10.3390/ijms252313061

**Published:** 2024-12-05

**Authors:** Ziyue Wang, Lanxiang Li, Miao Li, Zhonghua Lu, Lihua Qin, Robert Konrad Naumann, Hong Wang

**Affiliations:** 1 The Institute of Biomedical and Health Engineering, Shenzhen Institute of Advanced Technology, Chinese Academy of Sciences, Shenzhen-Hong Kong Institute of Brain Science-Shenzhen Fundamental Research Institutions, Shenzhen 518055, Chinahong.wang@siat.ac.cn (H.W.); 2 Department of Anatomy and Histoembryology, School of Basic Medical Sciences, Peking University Health Science Center, Beijing 100191, China; 3 University of Chinese Academy of Sciences, Beijing 100049, China; 4 Department of Pathology and Pathophysiology, Faculty of Basic Medical Sciences, Kunming Medical University, Kunming 650500, China

**Keywords:** *Gabre*, GABA_A_ receptor, preoptic area, body temperature, homeostasis, heart rate

## Abstract

The preoptic area of the hypothalamus is critical for regulation of brain–body interaction, including circuits that control vital signs such as body temperature and heart rate. The preoptic area contains approximately 70 molecularly distinct cell types. The *Gabre* gene is expressed in a subset of preoptic area cell types. It encodes the GABA receptor ε-subunit, which is thought to confer resistance to anesthetics at the molecular level, but the function of *Gabre* cells in the brain remains largely unknown. We generated and have extensively characterized a *Gabre-cre* knock-in mouse line and used chemogenetic tools to interrogate the function of *Gabre* cells in the preoptic area. Comparison with macaque *GABRE* expression revealed the conserved character of *Gabre* cells in the preoptic area. In awake mice, we found that chemogenetic activation of *Gabre* neurons in the preoptic area reduced body temperature, whereas chemogenetic inhibition had no effect. Furthermore, chemogenetic inhibition of *Gabre* neurons in the preoptic area decreased the heart rate, whereas chemogenetic activation had no effect under isoflurane anesthesia. These findings suggest an important role of preoptic *Gabre* neurons in maintaining vital signs such as body temperature and heart rate during wakefulness and under anesthesia.

## 1. Introduction

The preoptic area, the hypothalamus and selected nuclei of the midbrain and hindbrain harbor neural circuits and cell types that are essential for survival. They regulate the homeostatic interaction of the brain with the internal organs of the body such as the heart, the vascular system, and the reproductive organs [1,2,3,4]. In particular, the preoptic region of the hypothalamus contains a wide range of cell types that control key survival functions such as thermoregulation, thirst, and sleep, as well as social behaviors such as mating, parental behavior, and aggression [5,6,7,8,9]. Indeed, a key goal of neuroscience is to link individual cell types to specific behaviors, with cell types now most commonly defined by comprehensive screens of mRNA expression patterns [10]. Defined in this way, the mouse preoptic region contains 43 types of inhibitory (GABAergic) neurons and 23 types of excitatory (glutamatergic) neurons [11]. However, the function of most of these cell types remains unknown.

The most common way to gain access to specific cell types is to design transgenic mouse lines by introducing stable modifications at a single gene locus. However, many currently used transgenic lines target genes that are broadly expressed in multiple cell types in the preoptic area. Thus, further refinement and design of mouse lines with highly selective expression is critical to examine the role of specific cell types in the preoptic region. By screening gene expression databases for markers expressed in specific nuclei of the preoptic region [12,13], we observed that the *Gabre* gene shows a distinct expression pattern in the preoptic region. *Gabre* is one of the 19 genes encoding GABA_A_ receptor subunits. While the α, β, and γ subunits have been extensively studied, relatively little is known about the ε-subunit encoded by the *Gabre* gene [14]. Interestingly, while most GABA_A_ receptor subunits are widely expressed in multiple brain regions [15,16], *Gabre* expression appears to be more dynamic and restricted to a limited number of brain nuclei, which may provide clues to understanding the function of *Gabre*-expressing neurons. 

*Gabre* was originally cloned from human tissues and its presence in the brain and peripheral organs was validated using Northern blots [17,18,19,20]. In addition, Whiting et al. [18] demonstrated strong *Gabre* expression in the arcuate nucleus of the hypothalamus (Arc) in squirrel monkeys. Subsequent studies in rodents delineated expression in several brain regions including the preoptic area, amygdala, and locus coeruleus [21,22], frequently showing co-expression of the ε-subunit with widely projecting neurotransmitter systems, including histamine, orexin, and noradrenaline [22,23,24], which are intricately involved in the regulation of sleep. 

Experiments using heterologous expression systems suggest that the ε-subunit confers a higher rate of desensitization and resistance to anesthetic action, or leads to a higher than typical level of constitutive activity [18,25,26,27,28,29], although not all studies have consistently observed the same effects, possibly due to differences in expression levels and experimental approaches. 

Using post hoc identification by RT-PCR, Sergeeva et al. [30] found that spontaneous inhibitory postsynaptic potentials in *Gabre*-positive hypothalamic cells are resistant to propofol, while Kasparov et al., [31] showed that benzodiazepine insensitivity correlates with *Gabre* expression in the solitary tract nucleus. In pregnant rats, *Gabre* mRNA levels are upregulated in brain nuclei critical for respiratory function, suggesting dynamic expression of GABA_A_ receptor ε-subunits upon changes in neurosteroid levels [32]. Finally, recent work with patients has shown that decreased GABRE expression correlates with cognitive impairment in schizophrenia [33], while variants of the GABRE gene are associated with epilepsy [34,35]. In summary, the ε-subunit may confer anesthesia resistance at the molecular level and may play a role in several disease phenotypes, but little is known about the cellular or circuit function of neurons expressing *Gabre*. 

The aim of this study is to contribute to understanding the function of neurons expressing the *Gabre* gene in the medial preoptic area of the hypothalamus (mPOA^GABRE^ neurons). We generated and validated a *Gabre-cre* knock-in mouse line and used chemogenetic tools to interrogate the function of mPOA^GABRE^ neurons. We conclude that mPOA^GABRE^ neurons are involved in maintaining the vital signs and provide a novel target for selective manipulation of vital functions in awake animals and during anesthesia.

## 2. Results

### 2.1. Targeting Construct and Strategy to Generate Gabre-cre Mice

**Figure 1:**
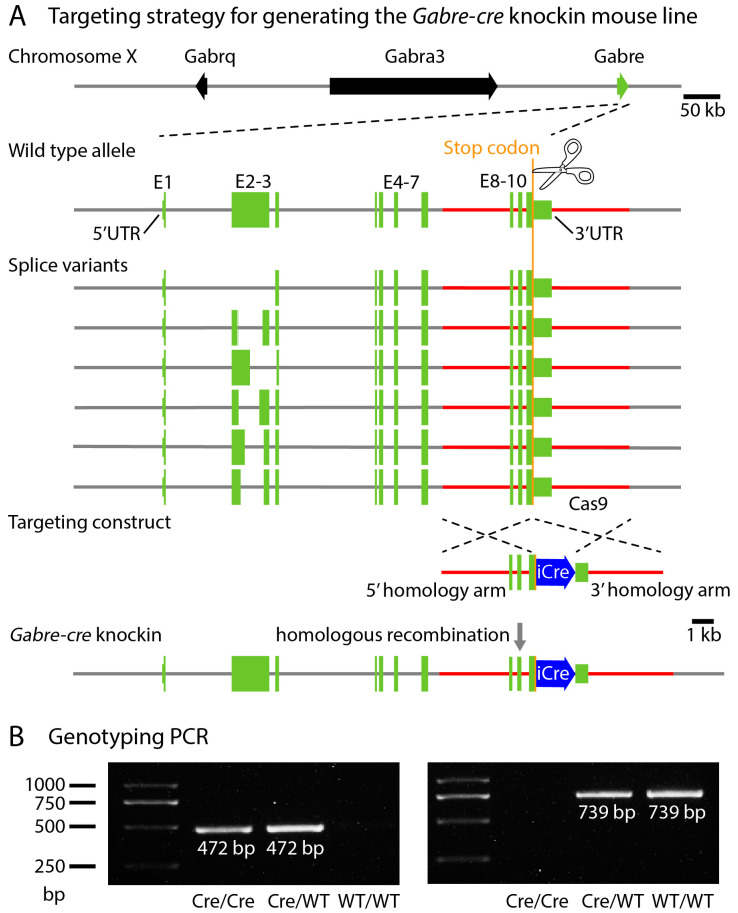
Generation of a *Gabre-cre* knock-in mouse line. (**A**) Targeting strategy. Open reading frames are mapped to chromosome X and illustrated with arrows and rectangles for *Gabre* (green) and iCre (blue). Six different splice variants were detected, mainly in exon 2. With CRISPR/Cas9 technology, the ires-iCre sequence was inserted 3′ to the *Gabre* stop codon. (**B**) PCR Screening. Genotyping primers used are described in the methods section. P1 + P3 detect the wild-type allele (739 bp), while P2 + P3 detect the *Gabre-cre* knock-in allele (472 bp).

The genes encoding the ε, α3, and θ subunits of the GABA_A_ receptor are located in tandem on the X chromosome (Figure 1A; [36]). To gain genetic access to cells expressing GABA_A_ receptors containing the ε-subunit, we designed a knock-in strategy targeting the *Gabre* gene. To identify splice variants, we extracted RNA from tissue of the preoptic area of postnatal day 14 mice. Following RNA isolation, cDNA synthesis, and sequencing, we found six splice variants of the *Gabre* gene, all located in exon 2 (Figure 1A). Exon 2 encodes repetitive sequences rich in proline and glutamic acid, which are absent in the human *Gabre* ortholog. To label cells expressing all variants, the ires-cre cassette was inserted 3′ of the stop codon using CRISPR/Cas9 technology. We designed specific genotyping primers to identify the Cre allele (Figure 1B). The mutant and wildtype PCR products were 739 bp and 472 bp long, respectively (Figure 1B).

### 2.2. Expression Analysis of Gabre-cre Mediated Recombination

**Figure 2:**
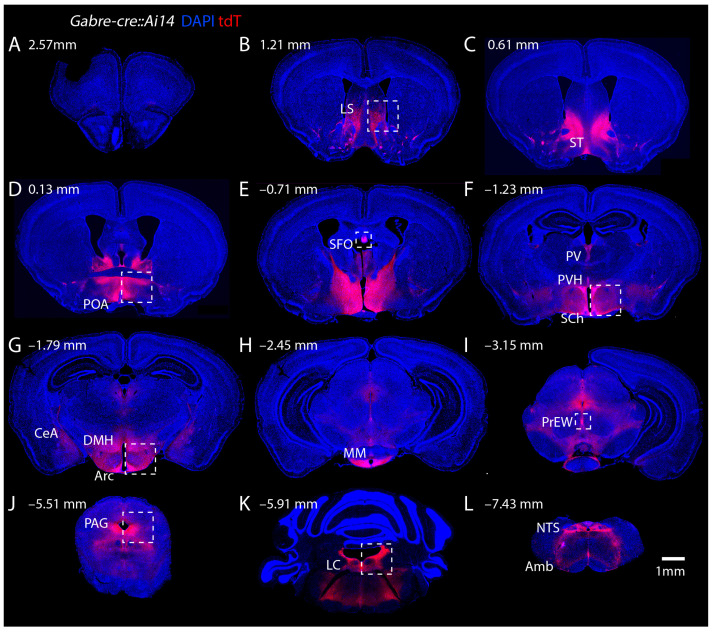
Cre-mediated tdT expression (red) in adult brains of *Gabre-cre::Ai14* mice. DAPI (blue) stained 50 μm coronal sections, shown from anterior (**A**) to posterior (**L**). Sections were selected to highlight regions with dense tdT expression in specific nuclei. The numbers represent anterior-posterior coordinates relative to bregma. (**A**–**L**) The most significant tdT signals in anterior sections are in the LS and BST whereas only few cells are found in the cortex. The POA and several hypothalamic nuclei such as the PVH, SCh, Arc, DMH, and MM densely express tdT. In addition, tdT is expressed in the SFO, PV, and PrEW regions. In posterior parts of the brain, we find tdT expression in the PAG, LC, NTS, and Amb. For abbreviations, see Table 1. Dashed boxes indicate regions with magnified images in Figure 3.

To identify the *Gabre-cre* mediated recombination, we crossed *Gabre-cre* mice with the Ai14 reporter line to induce tdTomato (tdT) fluorescence in *Gabre-cre* positive cells. We observed robust expression in subcortical areas (Figure 2). Overall, only very few neurons in the cerebral cortex expressed tdT (Figure 2A–I). In the anterior part of the brain, we detected strong fluorescent signals in the lateral septum (LS) and bed nucleus of the stria terminalis (BST) (Figure 2B,C). At the level of the anterior commissure, we observed recombination in the POA (Figure 2D) and more posteriorly in the subfornical organ (SFO) (Figure 2E). At the level of the tuberal hypothalamus, we detected tdT signals in the paraventricular thalamic nucleus (PV), the periventricular hypothalamic nucleus (PVH), the suprachiasmatic nucleus (SCh) (Figure 2F), the arcuate nucleus (Arc), the dorsomedial hypothalamic nucleus (DMH), and the medial mammillary nucleus (MM) (Figure 2G,H). The pre-Edinger–Wesphal nucleus expressed tdT (Figure 2I) and more posteriorly, the PAG had significant tdT signals ventral to the aqueduct (Figure 2J). We did not observe fluorescence in the cerebellum, but there were strong tdT signals in the locus coeruleus (LC) and Barrington’s nucleus (Bar) (Figure 2K). In the brainstem, the nucleus of the solitary tract (NTS) and the nucleus ambiguus (Amb) also showed strong tdT signals (Figure 2L).

**Figure 3:**
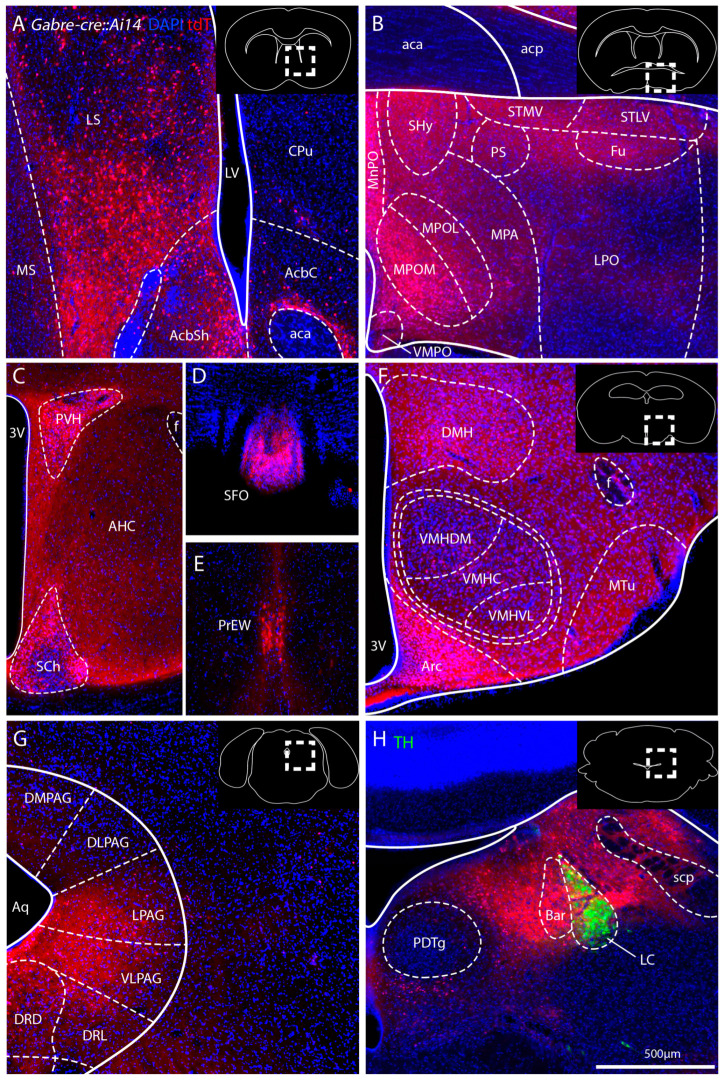
Higher resolution images of selected regions of *Gabre-cre::Ai14* mice. The locations are highlighted by a dashed box in schematic black and white images; see also Figure 2. (**A**) Dense labeled cells are located mainly in the ventral lateral septum. (**B**) Strong tdT signals detected in several nuclei of the preoptic area with varying intensity, among which the MPOM and MnPO show the highest density. (**C**) In the anterior hypothalamus, the PVH and the shell region of the SCh show a large number of labeled cells. (**D**,**E**) Dense labeling in the SFO and PrEW. (**F**) Strong tdT labeling is present around the 3 V. The Arc has a large number of labeled neurons, while the DMH and the MTu have a moderate density. (**G**) The PAG has tdT positive cells, mostly within the lateral and ventrolateral parts. (**H**) The LC and the Bar both express tdT. The green fluorescent signals represent tyrosine hydroxylase (TH) antibody staining. For abbreviations, see Table 1.

To further characterize *Gabre-cre* mediated recombination, we present several brain areas at higher resolution (Figure 3). We find labeled cells in the ventral part of the lateral septum (LS) and parts of the nucleus accumbens (Figure 3A). We observe tdT-expressing cells in several nuclei of the preoptic area (Figure 3B), including the median preoptic nucleus (MnPO), the periventricular hypothalamic nucleus (Pe) and the medial part of medial preoptic nucleus (MPOM). In addition, there are tdT-labeled fibers in the septohypothalamic nucleus (SHy), the lateral part of the medial preoptic nucleus (MPOL), the medial preoptic area (MPA), the parastrial nucleus (PS), the bed nucleus of the stria terminalis, as well as the ventromedial preoptic nucleus (VMPO). In contrast, the lateral preoptic area (LPO) shows little tdT expression. In the anterior part of the hypothalamus, we find densely labeled cells in the paraventricular hypothalamic nucleus (PVH) and the suprachiasmatic nucleus (SCh), the latter having labeled cells mainly in the shell of the SCh, with the core region devoid of tdT-positive cells (Figure 3C). The central anterior hypothalamic nucleus (AHC) shows only diffuse tdT labeling. We find intense labeling in two small central nuclei, the subfornical organ (SFO) and the pre-Edinger–Westphal nucleus (PrEW) (Figure 3D,E). In more posterior parts of the hypothalamus, the arcuate nucleus (Arc) is rich in tdT-expressing cells, whereas the dorsomedial hypothalamic nucleus (DMH), the medial tuberal nucleus (MTu), and the ventromedial hypothalamic nucleus (VMH) contain fewer tdT-positive neurons (Figure 3F). The periaqueductal gray (PAG) also exhibits tdT expression, mostly within the lateral and ventrolateral parts (Figure 3G). In contrast, the dorsolateral and dorsomedial parts of the PAG have few labeled neurons. The dorsal raphe nucleus (DR) also shows a moderate density of labeled cells. Ventral to the cerebellum, tdT signals are prominent in the locus coeruleus (LC) and Barrington’s nucleus (Bar) (Figure 3H). We visualized LC neurons using tyrosine hydroxylase (TH) antibody staining. Interestingly, we found little overlap between TH (green) and tdT expression in the LC, indicating that *Gabre* may present a marker of a distinct cell type in the LC.

### 2.3. Colocalization of Cre with Gabre in Different Brain Regions

**Figure 4:**
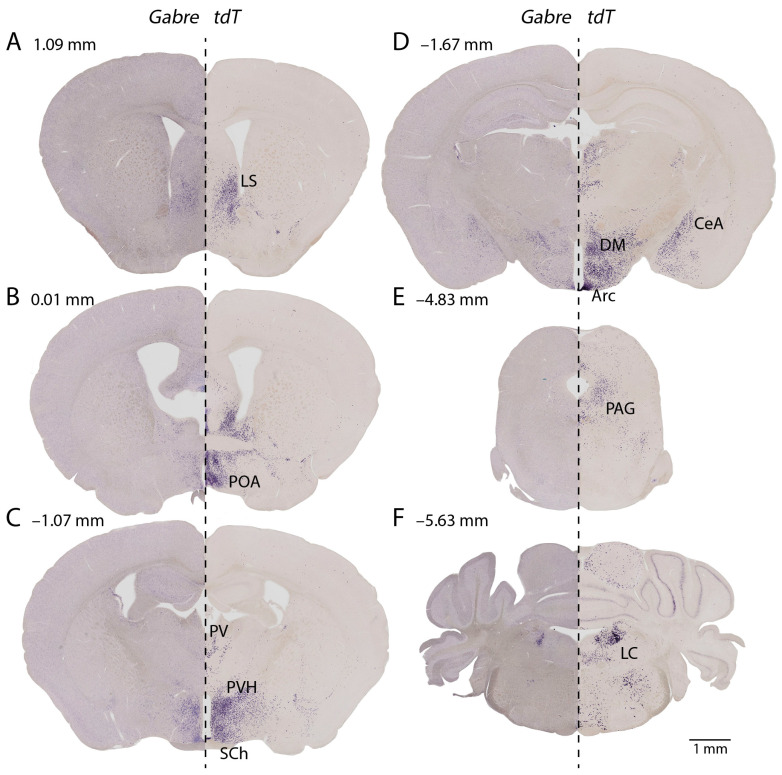
Comparison of *Gabre* (**left**) and *tdT* (**right**) ISH in *Gabre-cre::Ai14* mice. Sections are ordered from anterior (**A**) to posterior (**F**) with numbers indicating bregma coordinates. (**A**) The LS shows strong *tdT* signals and moderately strong *Gabre* expression. (**B**) The POA shows a high density of *tdT* signals; *Gabre* signals are moderate in the POA nuclei. (**C**) We detect strong *tdT* signals and few *Gabre* signals in the PV and with more *Gabre* in the PVH and SCh. (**D**) Large amounts of *tdT* labeled cells are detected in the CeA, DM, and Arc; however, *Gabre* expression is comparatively weaker. (**E**) We find moderately strong *tdT* signals in the PAG; however, *Gabre* shows little expression. (**F**) The LC shows dense *tdT* and *Gabre* expression. For abbreviations, see Table 1. See Table 2 for a semi-quantitative listing of expression levels in different brain regions.

To test whether *Gabre-cre* mediated recombination faithfully mirrored *Gabre* expression, we compared in situ hybridization (ISH) signals for *Gabre* and *tdT*, using the Ai14 reporter line crossed with *Gabre-cre* mice. We found that *Gabre* expression and *Gabre-cre* mediated recombination showed qualitatively similar expression patterns (Figure 4). Comparing the results, *tdT* signals generally covered the same brain regions as *Gabre* ISH signals but showed a higher signal-to-noise ratio. The LS displayed strong tdT signals and moderate expression of *Gabre* (Figure 4A). At the level of the anterior commissure, a large number of neurons showed *tdT* signals in the SHy, MnPO, medial preoptic nucleus (MPO), and Pe, whereas *Gabre* signals were weaker but present in these areas (Figure 4B). The PVH and SCh showed intense *tdT* expression but less *Gabre* expression (Figure 4C). Both *tdT* and *Gabre* were strongly stained in the ME, DMH, and Arc (Figure 4D). More posteriorly, we detected a moderate number of *tdT*-labeled cells in the PAG, while *Gabre* showed no expression, indicating transient expression during development (Figure 4E). The LC showed dense *tdT* signal and weaker staining in the Bar (Figure 4F). Similarly, *Gabre* showed significant signal in the LC, but almost none in the Bar (Figure 4F). Overall, the expression of *Gabre* and *tdT* was broadly comparable, although *tdT* signals typically showed a more uniform and somewhat broader expression level.

### 2.4. GABRE Expression in the Primate Brain

**Figure 5:**
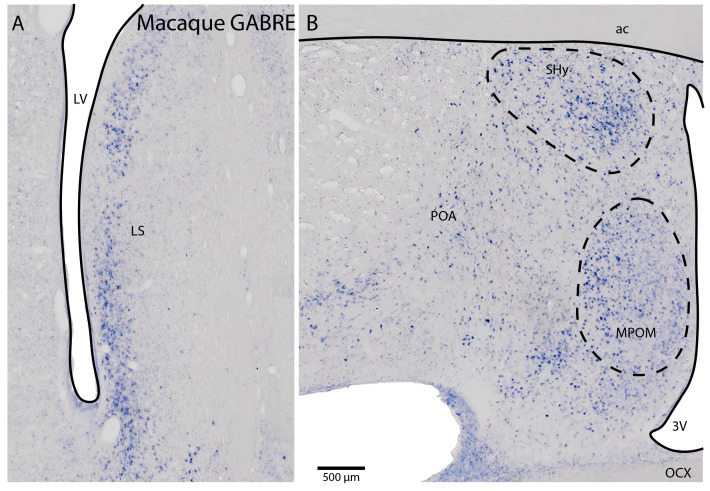
*GABRE* expression in macaque monkey LS and POA. (**A**,**B**). In situ hybridization for *GABRE* in the LS and POA regions of the macaque brain. For abbreviations, see Table 1.

Relatively little is known about *GABRE* expression in the non-human primate brain [18]. Therefore, we examined *GABRE* expression in macaque monkey brain sections. We found that *GABRE* had significant expression in the lateral septum (Figure 5A) and POA (Figure 5B). Interestingly, the SHy and MPOM subregions of the POA showed stronger expression than other nuclei in the POA, similar to the mouse *Gabre* expression, indicating evolutionary conserved expression patterns.

### 2.5. Chemogenetic Activation of mPOA^GABRE^ Neurons Decreases Body Temperature

**Figure 6:**
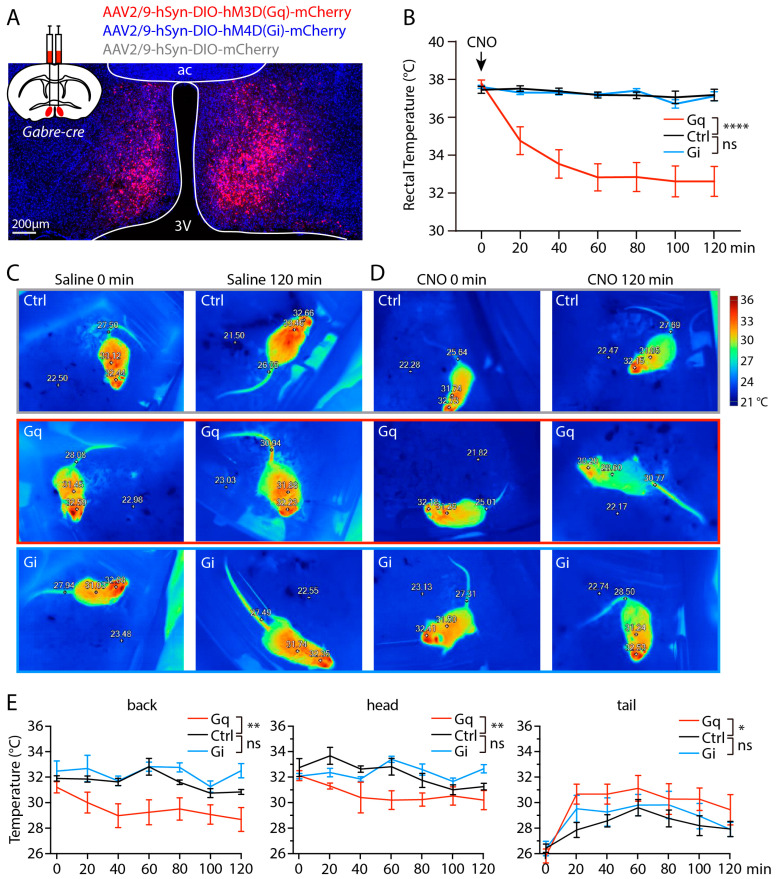
Chemogenetic activation of mPOA^GABRE^ neurons decreases body temperature. (**A**) Schematic figure and example of AAV injection targeting mPOA in the *Gabre-cre* mouse line. (**B**) Changes of rectal temperature after chemogenetic activation of mPOA^GABRE^ neurons (Gq, n = 7), chemogenetic inhibition (Gi, n = 6), and control group (Ctrl, n = 7). (**C**) Thermographs of mice injected with saline in Gq, Gi, and control groups. (**D**) Thermographs of mice injected with CNO in Gq, Gi, and control groups. (**E**) Quantification of surface temperature of the back, head, and tail regions after CNO injection in Gq (n = 6), Gi (n = 6), and control (n = 10) groups. The gray, red, and blue boxes in (**C**,**D**) indicate the Ctrl, Gq, and Gi groups, respectively. Asterisks indicate statistical significance (ns = not significant, * *p* < 0.05, ** *p* < 0.01, **** *p* < 0.0001). Two-way ANOVA was used to compare Gq with control and Gi with control groups, respectively. Data are shown as mean ± SEM.

Since the preoptic area is a key regulatory center for the control of temperature homeostasis [37], we wondered whether manipulating the activity of *Gabre*-positive neurons in the preoptic area (mPOA^GABRE^ neurons) would affect body temperature. To investigate the function of mPOA^GABRE^ neurons, we used bilateral delivery of AAVs encoding cre-dependent designer receptors exclusively activated by designer drugs (DREADD, [38]). We used AAVs encoding hM3D(Gq)-mCherry to activate and hM4D(Gi)-mCherry to inhibit mPOA^GABRE^ activity, and AAVs encoding mCherry as controls (Figure 6A). Clozapine-N-oxide (CNO), the cognate ligand of DREADD, was administered intraperitoneally (i.p.) at a dose of 3 mg/kg. Chemogenetic activation of mPOA^GABRE^ neurons significantly decreased rectal temperature (Figure 6B, Gq group, n = 7, control group, n = 7, 2-way ANOVA, *p* < 0.0001), while chemogenetic inhibition of mPOA^GABRE^ neurons had no effect on rectal temperature (Figure 6B, Gi group, n = 6, control group, n = 7, 2-way ANOVA, *p* = 0.717). CNO did not alter the rectal temperature of control animals. Using an infrared camera, we measured body surface temperatures in all groups. Images shown represent 0 and 2 h after injection of saline or CNO. Saline injection had no effect on surface temperature of Gq, Gi, or control group mice (Figure 6C). Two hours after CNO injection, only mice in the Gq group displayed a decrease in body surface temperature, while Gi and control group mice showed no change (Figure 6D). We analyzed the surface temperature of the back, head, and tail regions of the mice and detected a significant decrease of back and head surface temperature in Gq group compared with the control (Figure 6E, back temperature, Gq group, n = 6, control group n = 10, two-way ANOVA, *p* = 0.0046; head temperature, Gq group, n = 6, control group n = 10, two-way ANOVA, *p* = 0.0024). In the Gi group, the surface temperatures of both the back and head regions were comparable with the control group (Figure 6E, back temp, Gi group, n = 6, control group n = 10, two-way ANOVA, *p* = 0.784; head temp, Gi group, n = 6, control group n = 10, two-way ANOVA, *p* = 0.0513). In contrast to the drop of surface temperatures of the back and head regions, the tail temperatures were significantly higher in the Gq group compared with the control (Figure 6E, Gq group, n = 6, control group n = 10, 2-way ANOVA, *p* = 0.00357). Consistent with the unaltered core body temperature and surface temperatures of the back and head, the tail temperatures of the Gi group were similar to the control (Gi group, n = 6, control group n = 10, two-way ANOVA, *p* = 0.3780).

In summary, chemogenetic activation of mPOA^GABRE^ neurons robustly decreased core body temperature, while chemogenetic inhibition of mPOA^GABRE^ neurons did not affect body temperature (Figure 6B). The vasodilation of the tail presumably contributed to the heat loss after chemogenetic activation of mPOA^GABRE^ neurons (Figure 6E).

### 2.6. Chemogenetic Inhibition of mPOA^GABRE^ Neurons Decreases Heart Rate Under Isoflurane Anesthesia

**Figure 7:**
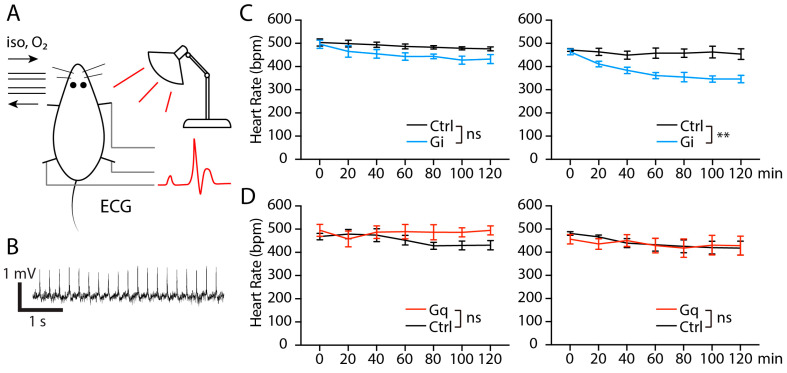
Chemogenetic inhibition of mPOA^GABRE^ neurons decreases heart rate under isoflurane anesthesia. (**A**) Schematic illustration of ECG recording during anesthesia. (**B**) A sample trace of the ECG recording. (**C**) CNO but not saline injection in the Gi group decreases heart rate. (Gi group, n =6; control group, n = 5, two-way ANOVA, *p* = 0.0834 for saline injection, *p* = 0.0030 for CNO injection). (**D**) Saline and CNO injections do not affect heart rate in the Gq group (Gq group, n = 5; control group, n = 5, two-way ANOVA, *p* = 0.2908 for saline injection, *p* = 0.1667 for CNO injection). Asterisks indicate statistical significance (ns = not significant, ** *p* < 0.01) using two-way ANOVA for comparison. Data are shown as mean ± SEM.

To reversibly manipulate activity of mPOA^GABRE^ neurons under anesthesia, we bilaterally injected Cre-dependent hM3D (Gq)-mCherry adeno-associated virus or hM4D (Gi)-mCherry adeno-associated virus into the mPOA of *Gabre-cre* mice. Mice were injected with CNO then anesthetized using an isoflurane gas mask with an oxygen flow at a rate of 200 ml/min under infrared light heating (Figure 7A). ECG was recorded with limb leads (Figure 7A,B). Anesthesia has a wide range of effects on the cardiovascular system but generally depresses cardiovascular function. However, isoflurane anesthesia preserves cardiac function better than other anesthetics [39]. Saline injections did not cause any significant change of heart rate in any experimental group. CNO injection in the hM4D (Gi) group induced a decrease in heart rate (*p* = 0.0030, 2-way ANOVA) compared with the control group (Figure 7C). However, CNO injection resulted in no significant alterations of heart rate in the hM3D (Gq) group compared with the control group (Figure 7D). In summary, inhibition of mPOA^GABRE^ neurons decreased the heart rate during anesthesia. Thus, our results suggest that manipulation of mPOA^GABRE^ neuron activity influences vital signs under anesthesia.

## 3. Discussion

We generated a novel mouse line, *Gabre-cre*, to study the expression and function of *Gabre*-expressing cells. Using *Gabre-cre::Ai14* mice, we characterized the detailed expression pattern of the *Gabre* gene in the entire mouse brain. Moreover, we report a conserved expression pattern of *Gabre* mRNA in the preoptic area of both macaque and mouse brains. Chemogenetic activation of mPOA^GABRE^ neurons decreased the body temperature in awake behaving mice. During general anesthesia, chemogenetic inhibition of mPOA^GABRE^ neurons reduced heart rate.

The expression of *Gabre* has been reported previously based on in situ hybridization and antibody staining [21,22,24]. Because the expression level of *Gabre* appears to be relatively low, only a few regions in the brain show consistent expression, namely the hypothalamus, locus coeruleus, and VTA [22,23,40,41]. In this study, we resolved the expression pattern of *Gabre* in the brain using genetic tools. We confirm the expression of *Gabre* in various hypothalamic nuclei, including the POA. In contrast to previous reports [17,20,42,43], we did not detect significant *Gabre* expression in the cerebellum, subthalamic nucleus, and cerebral cortex. However, we did not evaluate *Gabre* expression after repeated anesthesia, as was carried out in some of those studies. Sequeira and colleagues [44] reported GABRE expression in the human hypothalamus, although the exact subregion remains unknown. By studying brain sections of non-human primates, we determined the expression of *GABRE* in the medial preoptic area. The striking similarity of *Gabre* expression in mouse and macaque POA indicates a highly conserved regional expression pattern of *Gabre* and potential conserved function in controlling vital signs.

In mammals, body temperature is normally maintained around 37 °C and serves as an important vital sign, while deviations may indicate pathological conditions. The preoptic area receives and integrates information about body temperature, and manipulation of POA activity can reset body temperature to a different level [37,45]. This mechanism involves direct sensing of temperature in the POA by warm-sensitive neurons, which increase their firing rate upon warming and initiate heat loss mechanisms [46,47]. We found that chemogenetic activation of mPOA^GABRE^ neurons reduced both rectal and surface temperature. Thus, it is plausible that *Gabre* is expressed by warm-sensitive neurons. However, we previously showed that chemogenetic inhibition of POA^TRPM2^ neurons caused hyperthermia [5], while here, we found that inhibition of mPOA^GABRE^ neurons had no effect. Therefore, we speculate that *Gabre* may label a subset of *Trpm2*-expressing neurons and that increases and decreases in body temperature are regulated by different sets of POA neurons.

Although the brain under anesthesia is in general in a hypoactive state, some cells remain active to regulate the vital signs, such as respiration, cardiac activity, and temperature homeostasis [48,49]. Should these cells fail to function properly, complications such as hypothermia, cardiac arrhythmias, and hypoventilation may occur during general anesthesia. General anesthetics primarily target GABA_A_ receptors by binding to specific domains of the receptors [50]. Since GABA_A_ channels containing the ε-subunit cannot be potentiated by anesthetics in vitro, it is tempting to speculate that neurons expressing *Gabre* are part of a core network for maintaining vital functions during general anesthesia in vivo. The *Gabre*-containing neurons in the ventral respiratory column (VRC) constitute one example. Repeated exposure to general anesthetics increases *Gabre* expression in the VRC and renders phrenic nerve activity resistant to anesthesia [43]. During pregnancy, VRC neurons show resistance to general anesthetics compared with virgin and postpartum controls. This phenomenon coincides with the upregulation of *Gabre* in the VRC in pregnant rats [32]. Collectively, the expression of *Gabre* is necessary to confer resistance to general anesthetics. Over-expression of *Gabre* renders neurons insensitive to anesthetics both in vitro and in vivo [27,30,51]. Therefore, the presence of *Gabre* is also sufficient to confer resistance to general anesthesia.

A recent study [52] has shown that a subset of brainstem nucleus ambiguus neurons express *Gabre* and that activating these neurons can decrease the heart rate by 50% under isoflurane anesthesia. In addition, accumulating evidence indicates that unconsciousness induced by general anesthetics and sleep share common circuit elements in the preoptic area of the hypothalamus [53,54,55,56]. Activation of a specific group of POA neurons expressing bombesin-like receptor 3 raises both body temperature and heart rate [57], while chemogenetic stimulation of *Gabre*-expressing neurons in the POA reduces body temperature. This suggests that Brs3 and *Gabre* potentially label non-overlapping types of neurons. Interestingly, when mPOA^GABRE^ neurons are chemogenetically inhibited during anesthesia, the heart rate of the mice slows down by about 20%. This suggests that baseline activity of the mPOA^GABRE^ neurons is necessary to maintain the heart rate under anesthesia. Inhibition of mPOA^GABRE^ neurons may reduce baseline activity and further decrease the heart rate. Further studies are required to test whether *Gabre*-expressing neurons in the preoptic area represent a homeostatic control mechanism that maintains the vital signs under anesthesia.

Due to the unavailability of specific antibodies against Gabre, we could not quantify the expression of Gabre at the protein level and correlate it with the abundance of mRNA. Comparing the mammalian orthologs of *Gabre*, we noticed that an insertion of repetitive sequences encoded by exon 2 is only present in mice and rats. Although we identified alternative transcripts of exon 2, whether this proline-rich sequence is indeed translated remains unknown. A specific antibody would be crucial to verify the protein expression.

It is worth noting that the preoptic area is not a uniform structure. Based on single-cell transcriptomic sequencing, about 70 different cell types can be identified [11]. Whether *Gabre*-positive cells represent a single or multiple cell types warrants further studies. Possibly due to its low expression level, the *Gabre* gene was not captured in this dataset [11]. Future single-cell trasncriptomic profiling of *Gabre-cre::Ai14* mice may answer this question.

We used both male and female mice in this study but did not monitor the estrous cycle stages of the female mice. *Gabre* expression changes with the estrous cycle in female mice and is reduced in testicular feminization in male mice [58]. In addition, estrogen receptors, which are dynamically expressed in the preoptic area during the estrous cycle, may contribute to sex differences in response to anesthesia [59]. Whether our current findings can be generalized to female mice of all estrous cycle stages remains to be studied.

In this study, we have focused on the function of *Gabre*-positive neurons in the POA; however, without direct measurement of mPOA^GABRE^ neuronal activity, the functional interpretation of the results remains preliminary and hypothesis-generating for future studies.

Our data indicate that the *Gabre-cre* mouse line faithfully recapitulates the expression of the *Gabre* gene in the brain. Chemogenetic activation of *Gabre-*positive neurons in the POA reduces body temperature in awake mice, whereas chemogenetic inhibition reduces the heart rate during anesthesia. Hence, we hypothesize that POA Gabre neurons play a key role in maintaining vital signs in awake and anesthetized animals. Further studies are needed to clarify the neural circuit mechanisms for maintaining body temperature and heart rate in awake animals and under general anesthesia.

## 4. Materials and Methods

### 4.1. Animals

All experimental procedures were performed according to the institutional guidelines on animal welfare and approved by the local institution in charge of experiments using animals (Animal Care and Use Committee at the Shenzhen Institute of Advanced Technology (SIAT), Chinese Academy of Sciences (CAS), China; permit number SIAT-IRB-171016-NS-WH-A0384). In total, 49 female and male mice were used in this study. C57BL/6J mice were obtained from Beijing Vital River Laboratory Animal Technology Co., Ltd (Beijing, China). The Ai14 reporter line was imported from the Jackson Laboratory (Bar Harbor, ME, USA).

Macaque monkey brain sections were collected from a 10-month-old male crab-eating macaque (n = 1, body weight 1 kg; obtained from Guangdong Landau Biotechnology Co., Ltd. (Guangzhou, China), that was sacrificed for an unrelated experiment (Animal Care and Use Committee at the Shenzhen Institute of Advanced Technology (SIAT), Chinese Academy of Sciences (CAS), China; permit number SIAT-IACUC-210326-NS-WH-A1881).

### 4.2. Generation of Gabre-cre Mice

The Gabre-ires-cre (*Gabre-cre*) knock-in mouse line was generated by Shanghai Model Organisms Center, Inc. by inserting an ires-cre cassette in the 3′UTR of the *Gabre* gene locus in order to mimic the endogenous expression of *Gabre*. The Cas9 mRNA, gRNAs, and donor vectors were microinjected into zygotes of C57BL/6J mice. The sequences of the insertion site, crRNAs, and the complete sequence of the ires-cre cassette are shown in the Supporting Information Tables S1–S3. The founder was confirmed with long-range PCRs. It was backcrossed for more than three generations with wild-type C57BL/6J mice before use in the experiments. All *Gabre-cre* mice were kept on a C57BL/6J background and heterozygous animals were used for experiments. The genotype of *Gabre-cre* animals was verified by PCR with the primers shown in Table 3.

### 4.3. Tissue Preparation

Reagents were bought from Sigma-Aldrich (St. Louis, MO, USA), unless otherwise noted. Brain tissue was prepared as follows. Using pentobarbital, mice were deeply anesthetized. We perfused animals with 1× phosphate buffered saline solution (PBS) and 4% paraformaldehyde (PFA) in 0.1 M phosphate buffer (PB). Subsequently, brains were dissected out and fixed again in PFA overnight. Before sectioning, mouse brains were cryoprotected using an ascending series of 10% and 30% sucrose solution in PB, each step lasting at least 24 h. We used O.C.T. Compound (Tissue-Tek^®^ Sakura Finetechnical Co., Ltd., Tokyo, Japan) for embedding brains and prepared 50 µm-thick floating sections on a freezing microtome.

### 4.4. In Situ Hybridization

In situ hybridization was performed as described previously [5,60]. The primers used for generating in situ probes are shown in Table 4. DIG-labeled riboprobes were used for hybridization on 50 µm free floating cryosections. Hybridization was performed overnight at 65 °C. Sections were washed at 65 °C twice in 2xSSC/50% formamide/0.1% N-lauroylsarcosine and twice in 2xSSC/0.1% N-lauroylsarcosine at 37 °C for 20 min and twice in 0.2xSSC/0.1% N-lauroylsarcosine at 37 °C for 20 min. Sections were blocked in MABT/10% goat serum/1% blocking reagent (cat# 11096176001, Roche, Basel, Switzerland), incubated overnight with sheep anti-DIG-AP (1:1000, cat# 11093274910, Roche, Basel, Switzerland). After washing, staining was performed using NBT/BCIP in NTMT until satisfactory intensity was reached. The staining reaction was stopped with 10 mM EDTA. Sections were washed, dehydrated, and mounted with Eukitt^®^ quick-hardening mounting medium (Sigma-Aldrich, St. Louis, MO, USA).

Mouse cDNA was synthetized from total brain RNA using EasyScript First-Strand cDNA Synthesis SuperMix (TransGen Biotech, Beijing, China). Desired DNA fragments were amplified by PCR (Phusion, NEB, Beverly, MA, USA). PCR fragments were individually cloned in pEASY-Blunt Zero backbone (TransGen Biotech, Beijing, China) and verified by sequencing. Antisense digoxigenin-labeled riboprobes were synthesized according to the protocol recommended by the manufacturer (cat# 11277073910, Roche, Basel, Switzerland).

### 4.5. Image Acquisition

Chromogenic stainings were imaged at a 10× or 20× magnification using a slide scanner BX61VS (Olympus, Tokyo, Japan), an inverted confocal microscope LSM 880 (Carl Zeiss GmbH, Jena, Germany), or an ApoTome microscope (Axio Imager 2, Carl Zeiss GmbH, Jena, Germany). The fluorescent images were acquired in monochrome and color maps were applied to the images post-acquisition. Post hoc linear brightness and contrast adjustment were applied uniformly to the images under analysis.

### 4.6. Immunohistochemistry

For combining *Gabre* expression and antibody staining, we performed immunohistochemistry on sections from *Gabre-cre::Ai14* reporter mice. Sections containing the locus coeruleus were stained with antibody against tyrosine hydroxylase (1:500, MB318, Sigma-Aldrich, St. Louis, MO, USA). Sections were blocked in 5% bovine serum albumin (BSA) in PBS 1 h at room temperature, and then transferred to the primary antibody in PBS with 1% BSA and 0.5% Triton X-100 (X100, Sigma-Aldrich, St. Louis, MO, USA) for incubation at 4 °C overnight. Sections were washed in PBS, followed by 2 h room-temperature incubation with Alexa Fluor 488 goat anti-rabbit secondary antibody (1:2000, Jackson ImmunoResearch, West Grove, PA, USA) and DAPI (1:2500, Solarbio, Beijing, China). After washing in PBS, sections were mounted and coverslipped with Fluoromount Aqueous Mounting Medium (F4680, Sigma-Aldrich, St. Louis, MO, USA).

### 4.7. Virus Injection and Chemogenetic Activation

We used Cre-dependent adeno-associated viral vectors (AAV9-hSyn-DIO-hM3D(Gq)-mCherry, Taitool, S0425-9, or AAV9-hSyn-DIO-hM4D(Gi)-mCherry, Taitool, S0193-9) to specifically express excitatory hM3D receptors or inhibitory hM4D receptors in mPOA^GABRE^ neurons and AAV9-hSyn-mCherry (S0240-9, Taitool Bioscience, Shanghai, China) to express mCherry in control mice. Clozapine-N-oxide (CNO, APExBIO, Shanghai, China) was freshly dissolved to 0.3 mg/ml in sterile saline. We used a dose of 3 mg/kg CNO for all experiments.

In this study, 3% isoflurane was used to induce anesthesia, and 1–1.5% isoflurane to maintain anesthesia. After checking the lack of response to a toe pinch, mice were put onto a stereotaxic frame (RWD), with eye ointment to protect the eyes. Each mouse received 100 nl virus injection per side, targeting mPOA, using a syringe (10 μL, 7635-01, Hamilton Company, Reno, NV, USA) and a microinjection syringe pump (Micro4, WPI, Sarasota, FL, USA). The injection coordinates relative to bregma were AP +0.50 mm; ML ±0.5 mm; DV −5.0 mm. After microinjection, the syringe was held in position for 10 min to avoid virus reflux. The wound was sutured and covered with lincomycin hydrochloride and lidocaine hydrochloride gel to prevent inflammation. Subsequent to reducing the isoflurane concentration to 0, mice were monitored until recovery from anesthesia.

### 4.8. Detection of Splice Variants

High-quality total RNA was isolated from wild-type P14 mouse POA tissue using a RNeasy Lipid Tissue Mini Kit (Cat# 74804, Qiagen, Valencia, CA, USA). Mouse cDNA was synthetized from total RNA using EasyScript First-Strand cDNA Synthesis SuperMix (cat# AE301-02, TransGen Biotech, Beijing, China). Subsequently, two DNA bands from cDNA PCR products were observed and extracted using a Zymoclean Gel DNA Recovery Kit (cat# D4001, Zymo Research, Irvine, CA, USA). DNA fragments were cloned using the pEASY-Blunt3 vector (cat# CB301-01, TransGen Biotech, Beijing, China) and confirmed by sequencing.

### 4.9. Thermal Image Acquisition and Rectal Temperature Measurement

We used an infrared camera (TiX580, FLUKE, Everett, WA, USA) to acquire images every 20 min after intraperitoneal administration of freshly prepared CNO or saline for 2 h. Data were analyzed using SmartView 4.1 software (FLUKE, Everett, WA, USA). Rectal temperatures were measured together with the thermographs at the same time intervals (RET-3, TH-5, Physitemp, Clifton, NJ, USA).

### 4.10. Heart Rate Measurement

Mice were anesthetized with 3% isoflurane (RWD Life Science, Shenzhen, China) in an induction chamber, followed by 1.5% isoflurane to maintain anesthesia, using a mask with 100% oxygen flowing at 200 ml/min. Under infrared light heating, the rectal temperatures of the animals were kept at 36 °C during the experiment. We used an analog–digital converter (BL-420N, TECHMAN, Chengdu, China) equipped with recording software (BL-420N, TECHMAN, Chengdu, China) to continuously record ECG for 2 h after injection of CNO or saline. The R-R intervals were analyzed with software (BL-420N, TECHMAN, Chengdu, China). The heart rate data were analyzed using Prism8 (GraphPad Software Inc., San Diego, CA, USA).

### 4.11. Statistical Analysis

All data were tested for normality and are shown as mean ± standard error of the mean. A *p*-value <0.05 was considered statistically significant for all experiments. A two-way ANOVA was performed to analyze the effect of the time after i.p. injection and virus type (hM3D-mCherry, hM4D-mCherry, or mCherry) on body temperature. We report the effect of virus type on body temperature after simple main effects analysis. A two-way ANOVA was performed to analyze the effect of the time after i.p. injection and virus type (hM3D-mCherry, hM4D-mCherry, or mCherry) on heart rate. We report the effect of virus type on heart rate after the simple main effects analysis. We report the significance as follows: ns = not significant; * *p* < 0.05; ** *p* < 0.01; *** *p* < 0.001; **** *p* < 0.0001.

## Figures and Tables

**Table 1:** Abbreviations.

Abbreviation	Full Name
aca	Anterior commissure, anterior part
AcbC	Accumbens nucleus, core region
AcbSh	Accumbens nucleus, shell region
acp	Anterior commissure, posterior limb
AHC	Anterior hypothalamic nucleus, central
Amb	Ambiguus nucleus
Aq	Aqueduct
Arc	Arcuate hypothalamic nucleus
Bar	Barrington’s nucleus
CeA	Central amygdalar nucleus
CPu	Caudate putamen
DLPAG	Dorsolateral periaqueductal gray
DMH	Dorsomedial hypothalamic nucleus
DMPAG	Dorsomedial periaqueductal gray
DRD	Dorsal raphe nucleus, ventral part
DRL	Dorsal raphe nucleus, lateral part
f	Fornix
Fu	Bed nucleus of the stria terminalis, fusiform part
LC	Locus coeruleus
LPAG	Lateral periaqueductal gray
LPO	Lateral preoptic area
LS	Lateral septum
LV	Lateral ventricle
MM	Medial mammillary nucleus
MnPO	Median preoptic nucleus
MPA	Medial preoptic area
MPOL	Medial preoptic nucleus, lateral part
MPOM	Medial preoptic nucleus, medial part
MS	Medial septum
MTu	Medial tuberal nucleus
NTS	Solitary tract nucleus
PAG	Periaqueductal gray
PDTg	Posterodosal tegmental nucleus
Pe	Periventricular hypothalamic nucleus
POA	Preoptic area
PrEW	Pre-Edinger–Westphal nucleus
PS	Parastrial nucleus
PV	Paraventricular thalamic nucleus
PVH	Paraventricular hypothalamic nucleus
SCh	Suprachiasmatic nucleus
scp	Superior cerebellar peduncle
SFO	Subfornical organ
SHy	Septohypothalamic nucleus
ST	Bed nucleus of the stria terminalis
STLV	Bed nucleus of the stria terminalis, lateral division, ventral part
STMV	Bed nucleus of the stria terminalis, medial division, ventral part
VLPAG	Ventrolateral periaqueductal gray
VMHC	Ventromedial hypothalamic nucleus, central part
VMHDM	Ventromedial hypothalamic nucleus, dorsomedial part
VMHVL	Ventromedial hypothalamic nucleus, ventrolateral part
VMPO	Ventromedial preoptic nucleus
3V	3rd ventricle
4V	4th ventricle

**Table 2:** Expression patterns across brain regions. Signal strength: − no signal, + weak signal, ++ medium signal, +++ strong signal.

Brain Region	*Gabre-cre::Ai14* tdT Cells	*Gabre-cre::Ai14* tdT Neuropil	*tdT* ISH Cells	*Gabre* ISH Cells
Cerebral Cortex				
Motor Cortex	+	−	+	−
Somatosensory Cortex	+	−	+	−
Intermediate Endopiriform Nucleus	+	+	+	−
Cerebral Nuclei				
Lateral Septum	+++	++	+++	++
Medial Septum	−	+	+	−
Medial Amygdalar Nucleus	+++	++	++	−
Anterior Amygdalar Area	+++	+	++	−
Intercalated Amygdalar Nucleus	+++	++	++	−
Central Amygdalar Nucleus	+++	++	+++	++
Nucleus Accumbens	+	+	+	−
Bed Nucleus of the Stria Terminalis	++	++	++	+
Thalamus				
Intergeniculate Leaflet	++	−	+	−
Lateral Geniculate Complex, Ventral Part	+	−	+	−
Medial Geniculate Nucleus, Medial	+	+	++	−
Suprageniculate Thalamic Nucleus	+	+	+	−
Paraventricular Thalamic Nucleus	+++	++	+	−
Intermediodorsal Thalamic Nucleus	+	−	+	−
Reuniens Thalamic Nucleus	+	+	+	−
Central Medial Thalamic Nucleus	+	+	+	−
Hypothalamus				
Median Eminence	++	+++	++	+
Arcuate Hypothalamic Nucleus	+++	+++	+++	+++
Medial Tuberal Nucleus	++	+++	+	+
Dorsomedial Hypothalamic Nucleus	+++	+++	+++	+++
Ventromedial Hypothalamic Nucleus	++	++	+++	+
Zona Incerta	+	+++	+	+
Lateral Hypothalamic Area	+++	+++	++	+
Periventricular Hypothalamic Nucleus	+++	+++	+	++
Parastrial Nucleus	+	++	+	+
Medial Preoptic Nucleus, Medial Part	+++	++	+++	++
Medial Preoptic Nucleus, Lateral Part	++	++	++	+
Ventromedial Preoptic Nucleus	+++	++	+++	++
Septohypothalamic Nucleus	+	++	++	++
Lateral Preoptic Area	+	++	+	-
Medial Preoptic Area	+	++	++	++
Median Preoptic Nucleus	+++	++	++	+
Posterior Hypothalamic Nucleus	++	+++	++	+
Medial Mamillary Nucleus	+	++	+	+
Paraventricular Hypothalamic Nucleus	++	++	+++	−
Subfornical Organ	+++	+++	+++	+
Suprachiasmatic Area	++	+	+++	+
Anterior Hypothalamic Nucleus	++	++	++	+
Midbrain				
Pariaqueductal Gray	+++	+++	++	+
Pre-Edinger–Westphal Nucleus	++	+	++	+
Mesencephalic Reticular Formation	−	++	+	−
Isthmic Reticular Formation	−	++	+	−
Pedunculotegmental Nucleus	−	+	+	−
Interpeduncular Nucleus, Rostral Subnucleus	+	+	+	−
Dorsal Raphe Nucleus	+	+	++	+
Hindbrain				
Locus Coeruleus	++	+++	+++	+++
Solitary Tract Nucleus	+++	+	+++	+
Intermediate Reticular Nucleus	−	+	++	+
Ambiguus Nucleus	+	+	++	−
Botzinger Complex	+	+	+	−
Raphe Pallidus Nucleus	++	−	++	+
Inferior Olive, Principal Nucleus	+	+	++	+
Area Postrema	+	+	+++	−
Fiber tracts				
Superior Cerebellar Peduncle	−	+	−	−

**Table 3:** Primers for genotyping *Gabre-cre* mice.

Primer	Sequence (5′→3′)
P1	TTCCAACCAATAGCCGTGCTAATG
P2	TGGACCAATGTGAACATAGTGATGAACTAC
P3	CTGCTATACTGTTGCTCTGTGCATTCTG

**Table 4:** Primers for in situ hybridization.

Primer	Sequence (5′→3′)
*Gabre* mouse forward	ATACTCGAGTTGACATCATCTTCCACCAGACCTG
*Gabre* mouse reverse	TGGTTGGAAGTTGGTAGACCTTTAGAGAAGC
*tdT* forward	ATGGTGAGCAAGGGCGAGGA
*tdT* reverse	GGCATGGACGAGCTGTACAAG
*GABRE* macaque forward	TCTTCAAGGAGCATCCGTGATGC
*GABRE* macaque reverse	GTGACAGTGGGCTCTTGGATAGCTTC

## Data Availability

All data necessary to support the paper’s conclusions are presented in the main text.

## Supplementary Materials

The following supporting information can be downloaded at https://www.mdpi.com/article/10.3390/ijms252313061/s1.
