# Supplementary material for: Chemogenetic Modulation of Preoptic Gabre Neurons Decreases Body Temperature and Heart Rate
Source: Int J Mol Sci. 2024 Dec 5;25(23):13061. doi: 10.3390/ijms252313061 (PMC11641399; doi:10.3390/ijms252313061)
Supplement: Supplementary file 1 [file ijms-25-13061-s001.zip › ijms-3261415-supplementary.pdf]

Table S1. Flanking sequence of the insertion site, wild-type mouse genomic sequence.

GATAACTATTCGCGGGTGCTTTTTCCCATTACATTCTTCTTCTTTAATGTGCTCTAC
TGGCTGATTTGCCTTAACCTGTAGGCTCCAGCCAGTAGCTCATGGAGCAACCACC
TCAGTTCC

white background = 5′ flanking sequence >> red background = stop codon >> blue background = 3′ flanking sequence

Table S2. crRNA sequences.

crRNA	Sequence
crRNA1	GCTGATTTGCCTTAACCTGTAGG
crRNA2	AGCTACTGGCTGGAGCCTACAGG

Table S3. Recombined sequence.

GATAACTATTCGCGGGTGCTTTTTCCCATTACATTCTTCTTCTTTAATGTGCTCTAC
TGGCTGATTTGCCTTAACCTGTAGAGCAGGGCAAATGGATGTGTACTAGTTGAG
ATATCATCCCCGGGCCCCTCTCCCTCCCCCCCCCCTAACGTTACTGGCCGAAGCC
GCTTGGAATAAGGCCGGTGTGCGTTTGTCTATATGTTATTTTCCACCATATTGCCG
TCTTTTGGCAATGTGAGGGCCCGGAAACCTGGCCCTGTCTTCTTGACGAGCATTC
CTAGGGGTCTTTCCCCTCTCGCCAAAGGAATGCAAGGTCTGTTGAATGTCGTGAA
GGAAGCAGTTCCTCTGGAAGCTTCTTGAAGACAAACAACGTCTGTAGCGACCCT
TTGCAGGCAGCGGAACCCCCCACCTGGCGACAGGTGCCTCTGCGGCCAAAAGC
CACGTGTATAAGATACACCTGCAAAGGCGGCACAACCCCAGTGCCACGTTGTGA
GTTGGATAGTTGTGGAAAGAGTCAAATGGCTCTCCTCAAGCGTATTCAACAAGG
GGCTGAAGGATGCCCAGAAGGTACCCCATTGTATGGGATCTGATCTGGGGCCTC
GGTGCACATGCTTTACATGTGTTTAGTCGAGGTTAAAAAAACGTCTAGGCCCCCC
GAACCACGGGGACGTGGTTTTCCTTTGAAAAACACGATGATAATATGGCCACAC
TCGAGATATCgccagcatggtgcccaagaagaagaggaaagtctccaacctgctgactgtgcaccaaaacctgcctgc
cctccctgtggatgccacctctgatgaagtcaggaagaacctgatggacatgttcagggacaggcaggccttctctgaacacac
ctggaagatgctcctgtctgtgtgcagatcctgggctgcctggtgcaagctgaacaacaggaaatggttccctgctgaacctga
ggatgtgagggactacctcctgtacctgcaagccagaggcctggctgtgaagaccatccaacagcacctgggccagctcaaca
tgctgcacaggagatctggcctgcctcgcccttctgactccaatgctgtgtccctggtgatgaggagaatcagaaaggagaatgt
ggatgctggggagagagccaagcaggccctggcctttgaacgcactgactttgaccaagtcagatccctgatggagaactctg
acagatgccaggacatcaggaacctggccttcctgggcattgcctacaacaccctgctgcgcattgccgaaattgccagaatca
gagtgaaggacatctcccgcaccgatggtgggagaatgctgatccacattggcaggaccaagaccctggtgtccacagctggt
gtggagaaggccctgtccctgggggttaccaagctggtggagagatggatctctgtgtctggtgtggctgatgaccccaacaa
ctacctgttctgccgggtcagaaagaatggtgtggctgccccttctgccacctcccaactgtccacccgggccctggaagggatc
tttgaggccacccaccgcctgatctatggtgccaaggatgactctgggcagagatacctggcctggtctggccactctgccaga
gtgggtgctgccagggacatggccagggctggtgtgtccatccctgaaatcatgcaggctggtggctggaccaatgtgaacat
agtgatgaactacatcagaaacctggactctgagactggggccatggtgaggctgctcgaggatggggactgaaactgaggg
aggagcaccagtaaGCTCCAGCCAGTAGCTCATGGAGCAACCACCTCAGTTCC

white background = 5′ flanking sequence >> red background = stop codon >> yellow background = IRES >> lower case, white background = Cre >> blue background = 3′ flanking sequence
